# pH‐Responsive Side Chains as a Tool to Control Aqueous Self‐Assembly Mechanisms

**DOI:** 10.1002/chem.201904284

**Published:** 2019-12-13

**Authors:** Kalathil K. Kartha, Felix Wendler, Tobias Rudolph, Philip Biehl, Gustavo Fernández, F. H. Schacher

**Affiliations:** ^1^ Organisch-Chemisches Institut Westfälische Wilhelms-Universität Münster Corrensstrasse 40 48149 Münster Germany; ^2^ Laboratory of Organic and Macromolecular Chemistry and Jena Center for Soft Matter (JCSM) Friedrich Schiller University Jena Humboldtstrasse 10 07743 Jena Germany

**Keywords:** amphiphiles, pi-conjugated systems, self-assembly, stimuli-responsive, supramolecular polymerization

## Abstract

pH‐Tunable nanoscale morphology and self‐assembly mechanism of a series of oligo(*p*‐phenyleneethynylene) (OPE)‐based bolaamphiphiles featuring poly(ethylene imine) (PEI) side chains of different length and degree of hydrolysis are described. Protonation and deprotonation of the PEI chains by changing the pH alters the hydrophilic/hydrophobic balance of the systems and, in turn, the strength of intermolecular interactions between the hydrophobic OPE moieties. Low pH values (3) lead to weak interaction between the OPEs and result in spherical nanoparticles, in which aggregation follows an isodesmic mechanism. In contrast, higher pH values (11) induce deprotonation of the polymer chains and lead to a stronger, cooperative aggregation into anisotropic nanostructures. Our results demonstrate that pH‐responsive chains can be exploited as a tool to tune self‐assembly mechanisms, which opens exciting possibilities to develop new stimuli‐responsive materials.

## Graphical abstract

**The pH‐responsive behavior** of poly(ethylene imine) side chains has been exploited to tune the aggregate morphology and self‐assembly mechanisms of bolaamphiphiles. Our results open up exciting possibilities for the development of new stimuli‐responsive materials (see scheme).

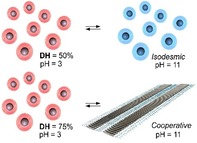

Supramolecular polymerization enables the creation of functional materials with multiple potential applications in various fields including optoelectronics or life sciences.^1^ This bottom‐up approach relies on the use of dynamic and reversible non‐covalent interactions, which provides the system with outstanding properties, such as responsiveness to external stimuli or self‐healing.^2^ Meanwhile, detailed mechanistic investigations of supramolecular polymerization processes have been reported for different types of building blocks.^3^ The majority of these systems can be described by two main polymerization mechanisms—isodesmic and cooperative.^4^ The determinants of a given mechanistic pathway are the type and number of non‐covalent interactions.^5^ Although supramolecular polymers only exhibiting π–π stacking are usually described by the isodesmic mechanism, the combination of π‐stacking and H‐bonding generally guarantees a cooperative mechanism, provided that no strong repulsive effects are present.^6^ Regulation of these mechanisms is a hot topic in current research, which is possible by altering the molecular design or by adjusting the physical parameters (temperature, solvent, and/or concentration) to prepare the aggregate solution. However, predicting the type of self‐assembly mechanism by molecular design is still challenging, and the development of advanced self‐assembled systems with tailored mechanisms is a prerequisite to generalize this trend.

Previously, our groups reported that the self‐assembly mechanism (isodesmic vs. cooperative) of polymer‐functionalized OPE‐based bolaamphiphiles in aqueous media can be directly correlated with the polymer chain length.^7^ This molecular engineering method enables the fine tuning of the hydrophilic/hydrophobic balance, which in turn determines the aggregate morphology (organized 2D lamellae vs. disorganized nanoparticles). These findings prompted us to investigate whether the hydrophilic/hydrophobic ratio and the resulting aggregation behavior could also be controlled by pH changes. In that regard, pH‐responsive polymer chains are an adequate tool to control charge and/or solubility by changes in pH.^8^ For this purpose, we have synthesized new pH‐responsive bolaamphiphilic systems (**1 a**,**b** in Scheme 1) composed of a linear OPE scaffold that is substituted on either end with water‐soluble poly(2‐ethyl‐2‐oxazoline) (PEtOx) side chains with a defined degree of polymerization (DP≈17). Additionally, structurally analogous systems with a lower DP of 12 (**2 a**,**b** in Scheme 1) have also been examined to broaden the scope of the approach. Partial hydrolysis of the PEtOx chains leads to linear poly(ethylenimine) (LPEI) units, which are well‐known weak polycations and thus able to respond to pH changes. Using established reaction conditions, the degree of hydrolysis could be adjusted quite precisely and determined by the comparison of the NMR signals correlating to the side chains and the PEI‐backbone signal in deuterated methanol.^9^ For the present systems under investigation, the side chains were partially hydrolyzed in a controlled manner to achieve degrees of hydrolysis (DH) of approximately 50 % (**1 a**,**2 a**) and 75 % (**1 b**,**2 b**). This results in a variable number of hydrophilic LPEI groups that can be protonated/deprotonated at appropriate pH values, which should affect the aqueous solubility of the polymer chains and therefore the strength of interactions between the hydrophobic OPE core. We hypothesize that the pH‐dependent degree of overlap of the OPE fragments might ultimately lead to a tunable self‐assembly mechanism and a different nanoscale morphology. At low pH, a higher degree of protonation of the LPEI units is expected to enhance the hydrophilicity of the system, as well as electrostatic repulsion between different bolaamphiphiles, and consequently, reduce the dimensionality of the aggregates in water. On the other hand, deprotonation of the LPEI groups can occur at high pH values, which may cause stronger hydrophobic interactions between OPE blocks and lead to more extended anisotropic structures (Scheme 1).

**Scheme 1:**
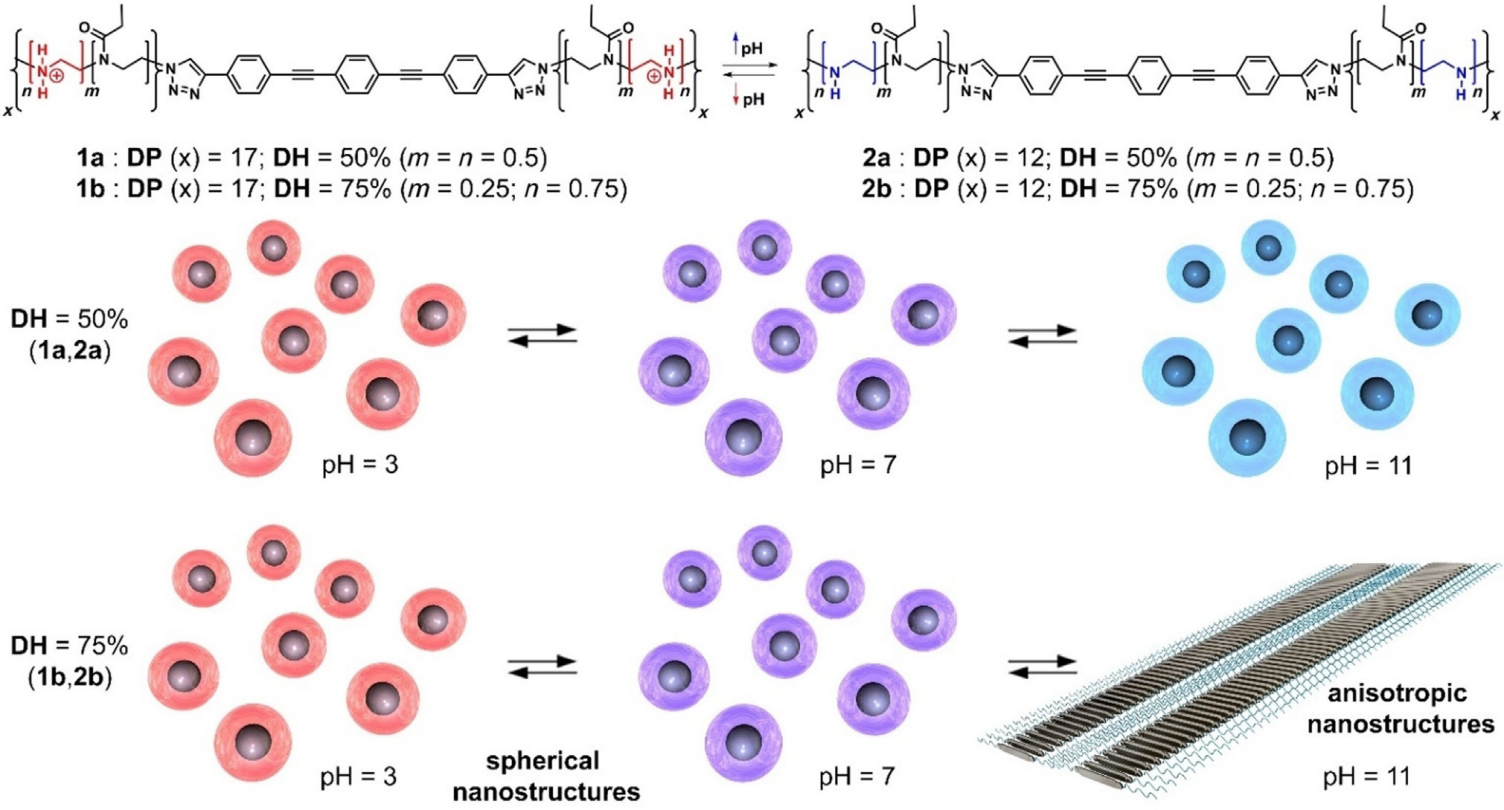
Chemical structures and composition of bolaamphiphiles **1 a**,**b** and **2 a**,**b** and schematic representation of their pH‐dependent nanoscale morphologies.

The target bolaamphiphilic systems **1 a**,**b** were synthesized according to a previously developed protocol (see the Supporting Information for details). Polymer chains with defined DH could be readily achieved by controlling the progression of the acidic hydrolysis in a microwave synthesizer in the presence of HCl (see the Supporting Information). Two defined DH of 50 % and 75 % were selected, which can be readily accessed by controlling the reaction time. The two bolaamphiphiles **1 a** and **b** with DP=17 and two different DH (≈50 % and 75 %) were screened to examine the effect of DH on the pH responsiveness. Initially, the sample with a DH of 50 % (**1 a**) was examined in a broad range of pH values (3–11). Regardless of the chosen pH value (3, 7, or 11), only clear solutions were observed without any noticeable turbidity or opacity, suggesting the absence of large aggregates that extend over two dimensions, such as 2D sheets or bundles of fibers (Figure 1 a). In sharp contrast, for **1 b**, increasing the number of groups that can be deprotonated under basic conditions leads to differences in the appearance of the solutions. Although the solutions at pH 3 and 7 remain transparent, a further pH increase to 11 leads to opaque solutions (Figure 1 b). The observations for **1 a** and **b** can be explained considering the number of hydrophilic repeat units (EtOx and (protonated) ethylenimine) of the polymer chains present at the respective pH values.

**Figure 1:**
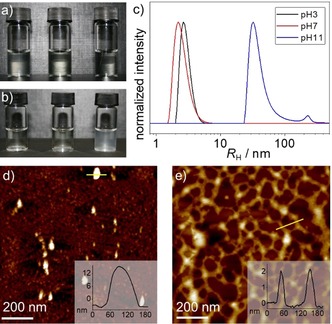
Photographs of the aqueous solutions (*c=*1.5×10^−4^ m) of **1 a** (a) and **1 b** (b) at different pH values (left to right: 3, 7, and 11). c) Number‐weighted DLS CONTIN plots of **1 b** (*c=*1.5×10^−4^ m) at pH values of 3, 7, and 11. AFM height images of **1 b** at pH 3 (d) and pH 11 (e) obtained by spin‐coating respective solutions (*c=*5×10^−6^ m) on HOPG; the insets show the height profile along the yellow lines.

For **2 b** (DH=50 %), the sum of EtOx units and the protonated PEI leads to 12 hydrophilic repeating units per side chain at pH 3, as all ethylene imine units are protonated. At pH 7, a DP of more than 8.9 can be assumed (Table S3 in the Supporting Information). In contrast, the number of hydrophilic repeating units is calculated as 5.8 at pH 11, which is below the critical value^7^ of six and anisotropic structures can be anticipated. For a DH of 75 % and an overall DP of 17 units per side chain (**1 b**), the calculated number of the hydrophilic repeating units gives a value of 6.6 at pH 11. In our opinion, a higher amount of uncharged ethylenimine units at this pH value is expected to increase the hydrophobicity, and consequently, the strength of π stacking of the individual OPE units.

To shed more light on the pH‐responsive behavior of **1 a**,**b**, the solutions at different pH values were investigated via dynamic light scattering (DLS), atomic force microscopy (AFM) and transmission electron microscopy (TEM). For **1 a**, DLS shows small particle sizes with hydrodynamic radii (*R*_h_) ranging from 1 to 10 nm (Figure S8 in the Supporting Information), demonstrating the absence of large aggregates regardless of the pH value. These results were expected considering that all solutions of **1 a** remained clear at all investigated pH values. Significantly different results were observed for the sample with a DH of 75 % (**1 b**). For this bolaamphiphile, DLS measurements yielded particle sizes in the range of *R*_h_=1–10 nm at pH of 3 and 7 (Figure 1 c). Interestingly, at pH 11, a main particle size distribution between approximately 22 and 150 nm with maximum at *R*_h_=32 nm, as well as a second minor contribution from 150 nm to 400 nm were observed (Figure 1 c). To gain further insights on the nanostructure formation, angular‐dependent light scattering studies have been recorded for the samples at pH 3 and 11 (Figure S9 in the Supporting Information). While a negligible angular dependency in DLS studies was observed for **1 b** at pH 3, significant changes in particle size are noticed at pH 11 depending on the measuring angle. These findings support the formation of isotropic structures, that is, spherical particles at pH 3 and anisotropic structures at pH 11 (Figure S9 in the Supporting Information).

The results extracted from DLS studies were further supported by AFM and (cryogenic) TEM imaging. As was expected, the images of a solution of **1 b** at pH 3 drop‐casted on a carbon‐coated copper grid show only small nanoparticles with sizes ranging from 10 to 20 nm in diameter (Figure S10 in the Supporting Information). In contrast, anisotropic structures with propensity to grow into 2D layered sheets were visualized at pH 11 (Figure S10). The different nanoscale morphology for the aggregates of **1 b** at pH 3 and pH 11 was further supported by AFM imaging upon spin coating the respective aggregate solutions onto highly oriented pyrolytic graphite (HOPG; Figure 1 d–e and S11 in the Supporting Information). In good agreement with TEM, nanoparticles with 10–20 nm height were observed for the sample at pH 3 (Figure 1 d). In contrast, a continuous network of 2D nanostructures with a uniform height of 2–4 nm and widths of 20–120 nm was visualized for **1 b** at pH 11 (Figure 1 e). Based on the overall results, we can conclude that only the self‐assembled structures formed by **1 b** are pH responsive, whereas **1 a** with lower DH is insensitive to pH changes. On this basis, in the following, we will exclusively focus on the mechanistic investigations of bolaamphiphile **1 b**.

To probe the influence of pH on the molecular packing and the resulting self‐assembly mechanism of **1 b** in aqueous media, absorption and emission studies were conducted. The aggregation was monitored in all cases by cooling monomer solutions at the respective pH values from 363 to 283 K by using a cooling rate of 0.2 K min^−1^. Regardless of the pH value, the molecularly dissolved state is achieved upon heating aqueous solutions of **1 b** (*c=*5×10^−6^ m) to 363 K. This was confirmed by comparing the normalized UV/Vis and fluorescence spectral signatures for all three samples at pH 3, 7, and 11 (Figure S12 in the Supporting Information). A gradual hypochromism of the monomeric absorption at 363 K was also observed upon varying the pH from 3 to 11 (0.54 to 0.44, see Figure 2), which might be due to slight differences in solvent quality for the monomer. To rule out potential aggregation effects as origin of the lower absorption intensity for the monomer at pH 11 compared to pH 3 and 7, additional DLS and UV/Vis experiments have been carried out (Figure S13 in the Supporting Information). Variable temperature (VT) UV/Vis studies of **1 b** at pH 3 showed minor spectral changes, that is, weak coupling of the OPE units^10^ upon cooling from 363 to 351 K, with negligible shifts of the absorption maximum (*λ*_max_=335 nm; Figure 2 a). Further cooling down to 283 K resulted in a slight increase of absorption with a small redshift in *λ*_max_ without any clear isosbestic point (Figure 2 a), which is in line with the formation of disorganized aggregates.^11^ Moreover, the formation of a weak aggregate shoulder band around 400 nm accompanied by a reduction of absorption around 270 nm are in accordance with the formation of small‐sized aggregates (Figure 2 a). The plot of the fraction of aggregated species (*α*_agg_) versus temperature derived from the VT UV/Vis experiments by monitoring at 335 nm has a sigmoidal shape, suggesting the existence of an isodesmic self‐assembly mechanism (Figure 2 a; inset). VT fluorescence studies of **1 b** at pH 3 showed an initial enhancement of the emission from 363 to 341 K, which can be ascribed to the restricted rotation and subsequent co‐planarization of the OPE backbone,^12^ a phenomenon that is often followed by aggregation. The subsequent weak quenching (20 %) that occurs upon further cooling from 341 to 283 K is in agreement with previous UV/Vis studies and supports the formation of small‐sized aggregates (Figure 2 b). Increasing the pH value to 7 leads to slightly different absorption and emission properties compared to pH 3. For example, VT UV/Vis experiments reveal a slight blue‐shift of 5 nm and an isosbestic point at 370 nm upon cooling (Figure 2 c), which contrasts with the slight red‐shift observed for the solution under more acidic pH values (see Figure 2 a). Additionally, the spectra show a single trend—a concomitant blueshift and slight decrease in absorption upon cooling from 363 K to 283 K, which contrasts with the small fluctuations in the absorption intensity observed at pH 3. VT emission studies show comparable results (initial increase of emission followed by quenching upon cooling) but, however, with a more significant fluorescence quenching (ca. 65 %) compared to pH 3 (Figure 2 d). The clearer trend observed in VT UV/Vis along with the more significant quenching of the fluorescence point to a slightly higher aggregation propensity of **1 b** at pH 7 than at pH 3. This is supported by the fact that the cooling curve (plot of *α*_agg_ vs. *T*) extracted from VT UV/Vis shows a more pronounced slope than at pH 3 (inset of Figure 2 a). However, the fact that both isodesmic and cooperative nucleation–elongation models are unable to describe this process reliably (see inset of Figure 2 c), indicates the lack of organized aggregates also at pH 7 (see Table S4 in the Supporting Information for further details). Notably, this behavior is completely different when the pH value was further increased to 11. VT UV/Vis studies show a rather significant sharpening and blueshift of *λ*_max_ from 335 to 315 nm upon cooling, along with an overall increase in absorption (Figure 2 e). In addition, a weak redshifted shoulder at 375 nm becomes more pronounced upon cooling. These spectral changes are typical of a face‐to‐face H‐type aggregation process, in which the stacked OPE units adopt a slightly twisted conformation,^13^ possibly to alleviate the steric effects caused by the bulky side chains. This behavior has been also reported for a number of π‐conjugated systems, including perylene bisimides,^14^ BODIPY dyes,^15^ and also OPEs.12a The formation of H‐type aggregates at pH 11 is further supported by the featureless and comparatively weaker emission band^16^ (quenching of ca. 80 %) upon cooling compared to lower pH values of 3 and 7 (Figure 2 f). On the basis of the spectroscopic results, we can conclude that the tendency of **1 b** to aggregate is considerably enhanced upon increasing the pH to 11, which might lead to a switch in the self‐assembly mechanism. In fact, the plot of *α*_agg_ vs. *T* derived from the VT UV/Vis experiments exhibits a clear non‐sigmoidal curve that is characteristic of a cooperative self‐assembly mechanism. Fitting of the cooling curve to the nucleation–elongation equilibrium model^17^ yields a high degree of cooperativity (*σ*) of 1.06×10^−5^ (Table S4 in the Supporting Information).

**Figure 2:**
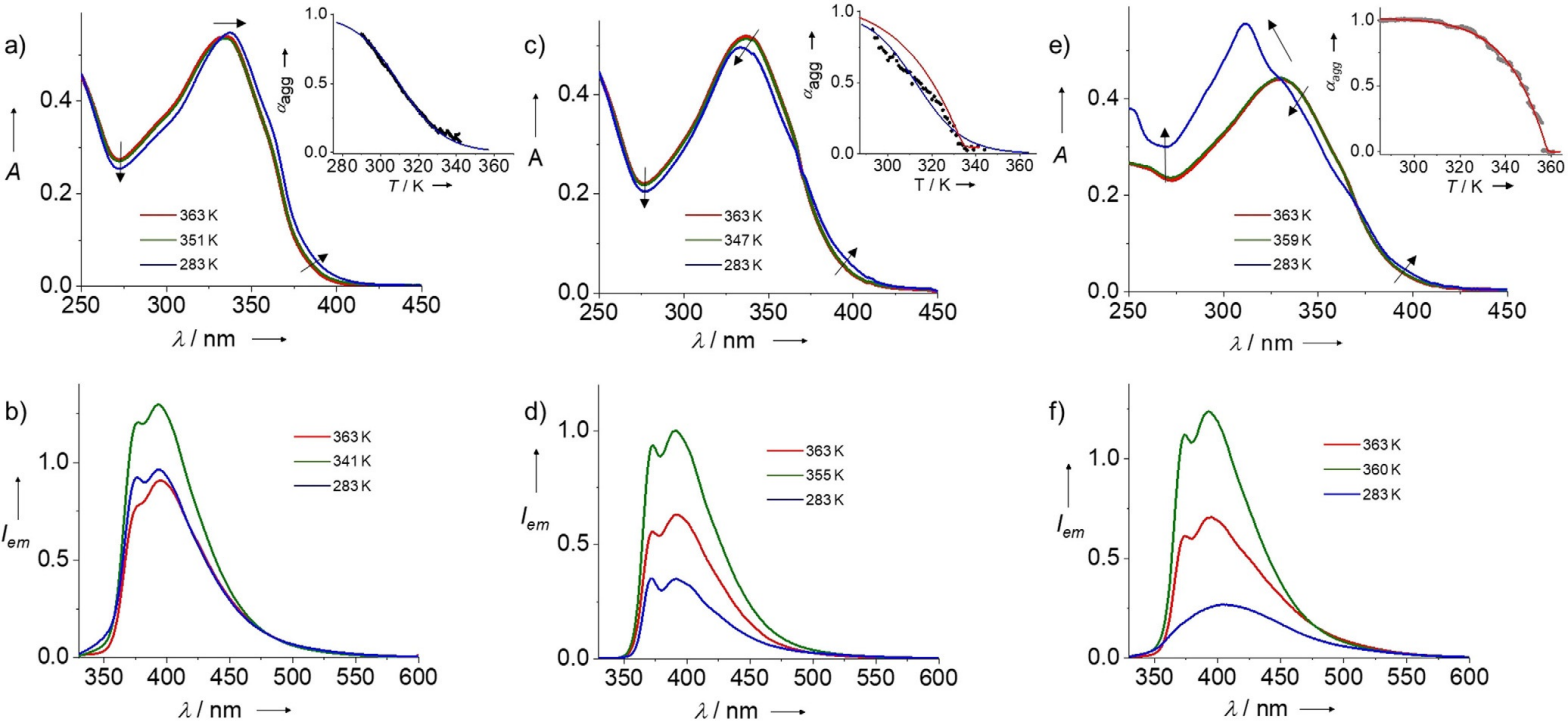
Variable temperature UV/Vis (a,c,e) and fluorescence (b,d,f) experiments of **1 b** (*c=*5×10^−6^ m) at pH 3 (a, b), pH 7 (c, d) and pH 11 (e, f); the insets show the corresponding cooling curves (plot of *α*_agg_ vs. temperature) obtained by monitoring the UV/Vis spectral changes at 335 nm (a,c) and 315 nm (e). The blue and red plots in the insets of Figures a,c and e represent the fits using the isodesmic (blue) and nucleation‐elongation cooperative model (red), respectively.

Comparing the overall results for both bolaamphiphiles **1 a** and **1 b**, we can extract the following conclusions: i) the bolaamphiphile with lower degree of hydrolysis (DH) (**1 a**: 50 %) has a very weak tendency to self‐associate regardless of the selected pH values (3, 7 or 11). For all pH values, small nanoparticles of 1–10 nm are formed as a result of a disorganized aggregation process; ii) increasing the DH to 75 %, as it is the case for **1 b**, leads to a higher number of ethylene imine groups that are susceptible of protonation, which provides the system with pH responsiveness. Thus, low pH values (3) lead to weak isodesmic aggregation into disorganized nanoparticles, similar to the behavior of **1 a** at all pH values. At pH 7, the aggregation propensity is slightly enhanced, but not to the point to switch the self‐assembly mechanism. This only occurs at the higher pH value of 11, where a clear cooperative H‐type self‐assembly process into anisotropic nanostructures is demonstrated.

To further validate the applicability of our approach, structurally analogous bolaamphiphiles **2 a**,**b** with shorter polymer chains (DP=12) and DH of 50 % and 75 % were synthesized and investigated (see S.I.). Remarkably, these bolaamphiphiles show an almost identical behavior compared with their respective homologues with longer chains: a relatively weak aggregation into disorganized nanoparticles for the sample with lower DH (50 %; **2 a**), regardless of the pH values (Figure S14). In sharp contrast, the bolaamphiphile with DH values of 75 % (**2 b**) shows, in analogy to **1 b**, a pH‐responsive aggregation behavior: the clear solutions observed at pH 3 and 7 strongly suggest the formation of small nanostructures, as also demonstrated by DLS and TEM (Figure S15). Raising the pH to 11 induces turbidity in the solution, which can be explained by a cooperative self‐assembly process into anisotropic nanostructures (Figure S16 and Table S4).

To conclude, we have unraveled the relationship between pH and the growth mechanism in self‐assembled systems. To this end, we have synthesized four bolaamphiphiles composed of a hydrophobic OPE‐based scaffold that is functionalized on both ends with polymer chains of different length (DP=12, 17) and degree of hydrolysis (DH=50, 75 %). Various studies bring to light that only the aggregates of those bolaamphiphiles with higher DPs of 75 % are responsive to pH changes in terms of altered self‐assembly mechanism. Low and medium pH values (3, 7) lead to weak aggregation and induce the formation of disorganized nanoparticles that follow the isodesmic mechanism. Interestingly, higher pH values (11) cause a much more significant aggregation into anisotropic nanostructures that are formed via the nucleation–elongation mechanism. Our results have allowed us to establish a relationship between the nanoscale morphology, self‐assembly mechanism, and pH, which paves the way for the development of advanced stimuli‐responsive self‐assembled systems with tunable mechanisms.

## Conflict of interest

The authors declare no conflict of interest.

## Supporting information

As a service to our authors and readers, this journal provides supporting information supplied by the authors. Such materials are peer reviewed and may be re‐organized for online delivery, but are not copy‐edited or typeset. Technical support issues arising from supporting information (other than missing files) should be addressed to the authors.

Supplementary
