# Supplementary material for: pH‐Responsive Side Chains as a Tool to Control Aqueous Self‐Assembly Mechanisms
Source: Chemistry. 2019 Dec 13;26(3):606–10. doi: 10.1002/chem.201904284 (PMC7003937; doi:10.1002/chem.201904284)
Supplement: Supplementary file 1 — Supplementary [file CHEM-26-606-s001.pdf]

CHEMISTRY
A European Journal

Supporting Information

pH-Responsive Side Chains as a Tool to Control Aqueous Self-Assembly Mechanisms

Kalathil K. Kartha+,[a] Felix Wendler+,[b] Tobias Rudolph,[b] Philip Biehl,[b] Gustavo Fernández,*[a] and F. H. Schacher*[b]

chem_201904284_sm_miscellaneous_information.pdf

Index

A. Description of Experimental Techniques **S3-S4**

General

Dynamic Light Scattering (DLS)

Transmission Electron Microscopy (TEM)

Optical Measurements

B. Synthesis and Characterization **S4-S10**

C. Supplementary Figures and table **S10-S15**

D. References **S15**

A. Description of Experimental Techniques

General. All solvents were dried according to standard procedures. Reagents were used as purchased. All starting materials were purchased from Sigma-Aldrich, Roth, ABCR, Merck or Acros Organics and were used as received, unless otherwise noted. 2-Ethyl-2-oxazoline (EtOx) and methyl tosylate (MeOTs) were distilled from barium oxide. 1,4-bis((4-ethynylphenyl)ethynyl)benzene (OPE) and PEtOx$_x$-N$_3$ were synthesized as described in our previous literature report[S1].

Dynamic Light Scattering (DLS)

Dynamic light scattering (DLS) was performed at a scattering angle of 90° on an ALV CGS-3 instrument equipped with a He-Ne laser operating at a wavelength of 633 nm at 25 °C. The CONTIN algorithm was applied to analyze the obtained correlation functions. For temperature control, the DLS is equipped with a Lauda thermostat. Apparent hydrodynamic radii were calculated according to the Stokes-Einstein equation.

Transmission Electron Microscopy (TEM)

The formed aggregates were analyzed using a transmission electron microscop (TEM) (Zeiss-CEM 902A, Oberkochen, Germany) operated at 80 kV. Images were recorded using a 1k TVIPS FastScan CCD camera. TEM samples were prepared by applying a drop of an aqueous sample solution onto the surface of a plasma-treated carbon coated copper grid.

Atomic Force Microscopy (AFM)

AFM images were recorded on a Multimode® 8 SPM System (AXS Bruker). Silicon cantilevers with a nominal spring constant of 9 Nm^{-1} and with resonant frequency of ~150 kHz and a typical tip radius of 7 nm (OMCL-AC200TS, Olympus) were employed.

Optical Measurements. Electronic absorption spectra were recorded on a Jasco UV–770-ST UV–Vis/NIR spectrophotometer and emission studies were performed on Jasco FP-8500 spectrofluorometer. All experiments were carried out using quartz cuvettes with optical pathlength of 1 cm. For all measurements, spectroscopic grade solvents (*Uvasol*) from *Merck* were used.

Sample preparation: To investigate the pH-responsiveness of **1a-b** and **2a-b** the materials were dissolved in the non-selective solvent mixture dimethyl sulfoxide (DMSO) – water (1.5:3.5). Afterwards, the samples were dialyzed against a buffer solution (disodium hydrogen phosphate 0.2M – citric acid 0.1M) and adjusted to a pH value of 7. PEI as a weak polyelectrolyte[S3] would strongly influence the pH and thus no representative and reproducible self-assembly might be investigated. To prevent this, buffer solutions were applied. Subsequently, one third was taken as such for the investigation of the self-assembly behavior in water. The other two-thirds were divided into two halves and further dialyzed against a buffer solution (glycine 0.1M/sodium chloride 0.1M – hydrochloric acid 0.1M) and adjusted to a pH value of 3 or dialyzed against a buffer solution (glycine 0.1M/sodium chloride 0.1M – sodium

hydroxide 0.1M) and adjusted to a pH value of 11. A similar concentration of 1 mg mL^{-1} for all samples was targeted.

DLS measurements with regard to the Stokes-Einstein equation following the assumption that the scattered particles are spherical. Non-spherical particles generally lead to discrepancies in size determinations.

TEM measurements: the samples were drop-casted on carbon-coated TEM grids. To avoid artefacts of the salt from the buffer solutions, 2 drops of pure water were applied to the TEM grid after plotting the sample drop, removing as much of the salt as possible.

Optical studies: All sample were diluted to the required concentration before UV-Vis and emission measurements. Absorption changes were recorded from 200 to 600 nm and emission changes were monitored by exciting at 300 nm and collecting from 330-700nm with a 1cm cuvette at 25 ºC. A hot solution of the samples at 363 K were cooled down to 283 K with a cooling rate of 0.2 K/min. Always freshly prepared buffer solutions were used for checking the reproducibility of the results.

AFM measurements: The aggregates were obtained in an identical way as for UV/Vis studies (slow cooling of the monomer solution). Then, 50-100 µL of solution was spin-coated onto highly oriented pyrolytic graphite (HOPG) with an rpm of 2000. The residual solvent evaporation was confirmed by leaving the samples at room temperature overnight.

SEC measurements: Size-exclusion chromatography was performed on a Shimadzu system equipped with a SCL-10A system controller, a LC-10AD pump, a RID-10A refractive index detector, a SPD-10A UV detector at 365 nm using a solvent mixture containing chloroform, triethylamine and isopropanol (94:4:2) at a flow rate of 1 mL min^{-1} on a PSS-SDV-linear M 5 µm column. The system was calibrated with polystyrene, poly(methyl methacrylate) and poly(ethylene oxide) standards.

MALDI-ToF-MS measurements: MALDI-ToF-MS spectra were measured on an Ultraflex III TOF/TOF (Bruker Daltonics GmbH) equipped with a Nd:YAG laser and a collision cell. All spectra were measured in the positive reflector or linear mode using DHB or DCTB as matrix.

FT-IR measurements: Fourier transform infrared spectroscopy was performed on a FT-IR spectrometer IR Affinity-1 from Shimadzu.

B. Synthesis and Characterization of 1a-b and 2a-b.

Synthesis of $PEtOx_x$-OPE-$PEtOx_x$

1,4-bis((4-ethynylphenyl)ethynyl)benzene (OPE, 20 mg, 0.06 mmol) and $PEtOx_x$-N_3 (0.13 mmol, 2.2 eq.) were dissolved in 10 mL THF. CuBr (5 eq.) and PMDETA (5 eq.) were added and the corresponding reaction solution was stirred for 1 hour at 50 °C. Subsequently, the solution was diluted with methylene chloride and the copper was removed by washing with water several times until no more coloration of the respective aqueous layer could be observed.

The organic layer was dried over sodium sulfate and the solvent was evaporated under reduced pressure. The residue was dissolved in 10 mL methylene chloride and subsequently the polymer was precipitated in cold diethyl ether. The resulting suspension was allowed to stand at room temperature overnight to get rid of the excess of unconverted homopolymer (soluble in warm ether). Subsequently, the yellowish suspension was centrifuged (5 min, 8000 rpm) and the residue washed with room tempered diethyl ether and dried in vacuo.

Characterization of PEtOx$_x$-OPE-PEtOx$_x$:

^{1}H NMR (300 MHz, $CDCl_3$, δ): 7.9-7.4 (aromatic), 3.6–3.1 (br, –N–*CH$_2$*–*CH$_2$*–), 2.5–2.1 (br, CO–*CH$_2$*–*CH$_3$*), 1.3–0.9 (br, CO–*CH$_2$*–*CH$_3$*) ppm.

^{1}H-NMR (300 MHz, DMSO, δ): 8.7-7.5 (aromatic), 3.6-3.0 (br, –N–*CH$_2$*–*CH$_2$*–), 2.4–2.1 (br, CO–*CH$_2$*–*CH$_3$*), 1.1–0.7 (br, CO–CH_2–*CH$_3$*) ppm.

^{13}C-NMR (75 MHz, DMSO, δ): 174-170 (*C*=O), 133-130 ($C_{aromatic}$H, $C_{triazol}$H), 125 ($C_{aromatic}$), 92-90 (*C*≡*C*), 47-42 (backbone), 26-24 (CO–CH_2–CH_3), 11-8 (CO–CH_2–CH_3) ppm.

IR: *v* [cm^{-1}] = 2970 and 2936 (CH), 2356 (C≡C), 1738 (-C_{ar}=C_{ar}), 1636 (N-C=O), 1426 (CH_2/CH_3), 1372 (CH_3), 1206 (C-N).

PEtOx$_{12}$-OPE-PEtOx$_{12}$: SEC ($CHCl_3$/*i*-PrOH/Et_3N): M_n = 3,000 g mol^{-1}; PDI = 1.07 (PS calibration), MS (MALDI-ToF, DCTB): M_p = 2 650 g mol^{-1}; yield: 33%.

PEtOx$_{17}$-OPE-PEtOx$_{17}$: SEC ($CHCl_3$/*i*-PrOH/Et_3N): M_n = 3,950 g mol^{-1}; PDI = 1.08 (PS calibration), MS (MALDI-ToF, DCTB): M_p = 3 600 g mol^{-1}; yield: 40%.

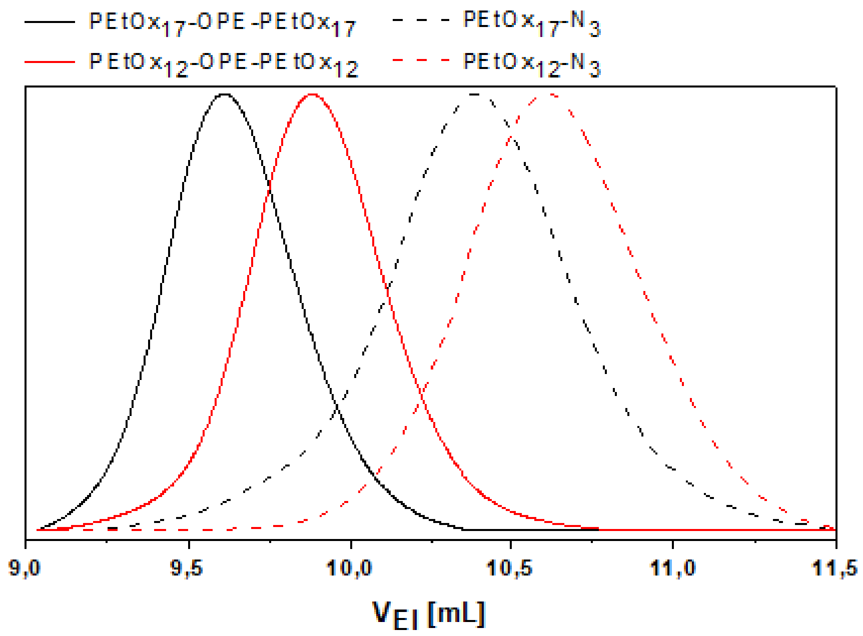

Figure S1: Comparison of SEC traces for the CuAAC click reaction between OPE and PEtOx$_{12/17}$-N_3 (black and red dashed lines) and their products PEtOx$_{12/17}$-OPE-PEtOx$_{12/17}$ (black and red straight lines).

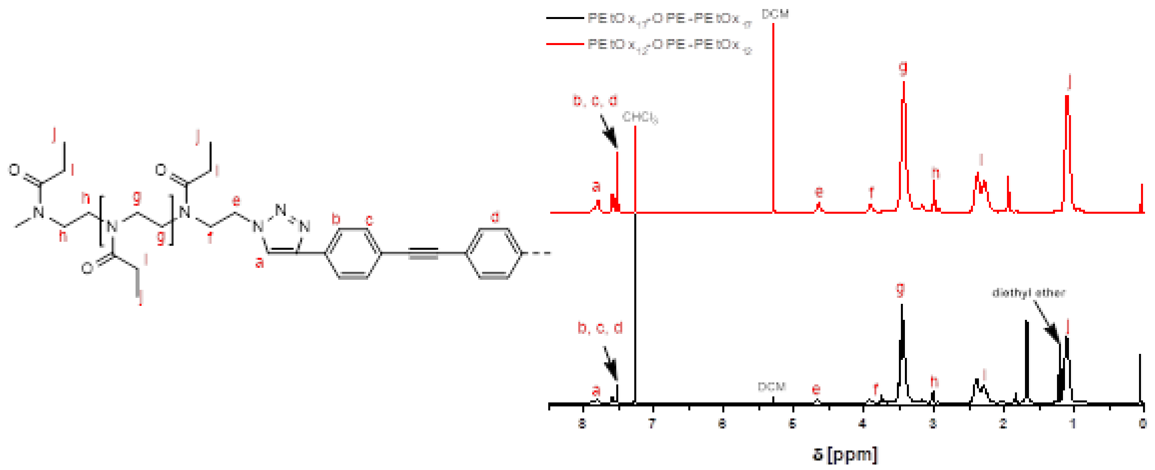

Figure S2: NMR characterization of PEtOx$_{12/17}$-OPE-PEtOx$_{12/17}$ via ^{1}H-NMR (300 MHz, $CDCl_3$).

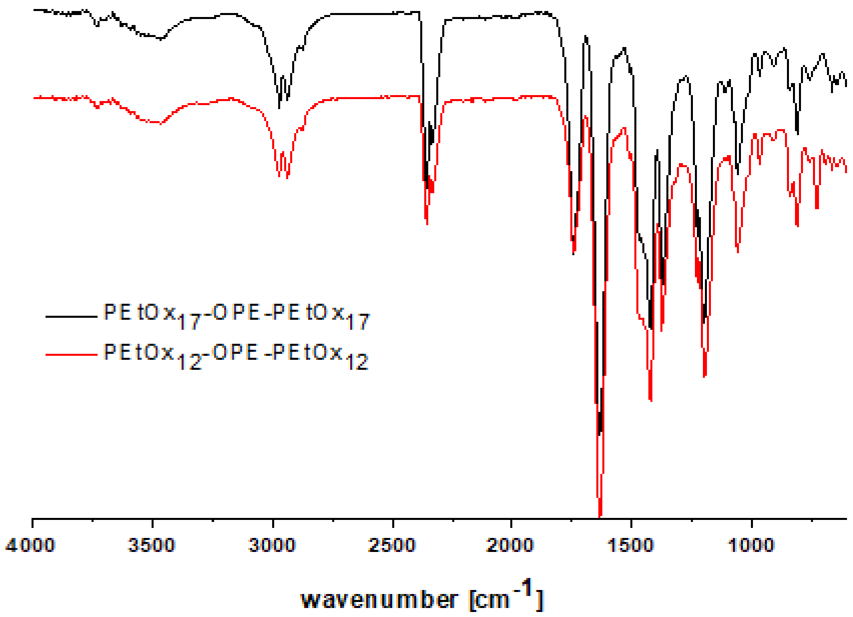

Figure S3: Comparison of FT-IR spectra for PEtOx$_{12}$-OPE-PEtOx$_{12}$ (red line) and PEtOx$_{17}$-OPE-PEtOx$_{17}$ (black line).

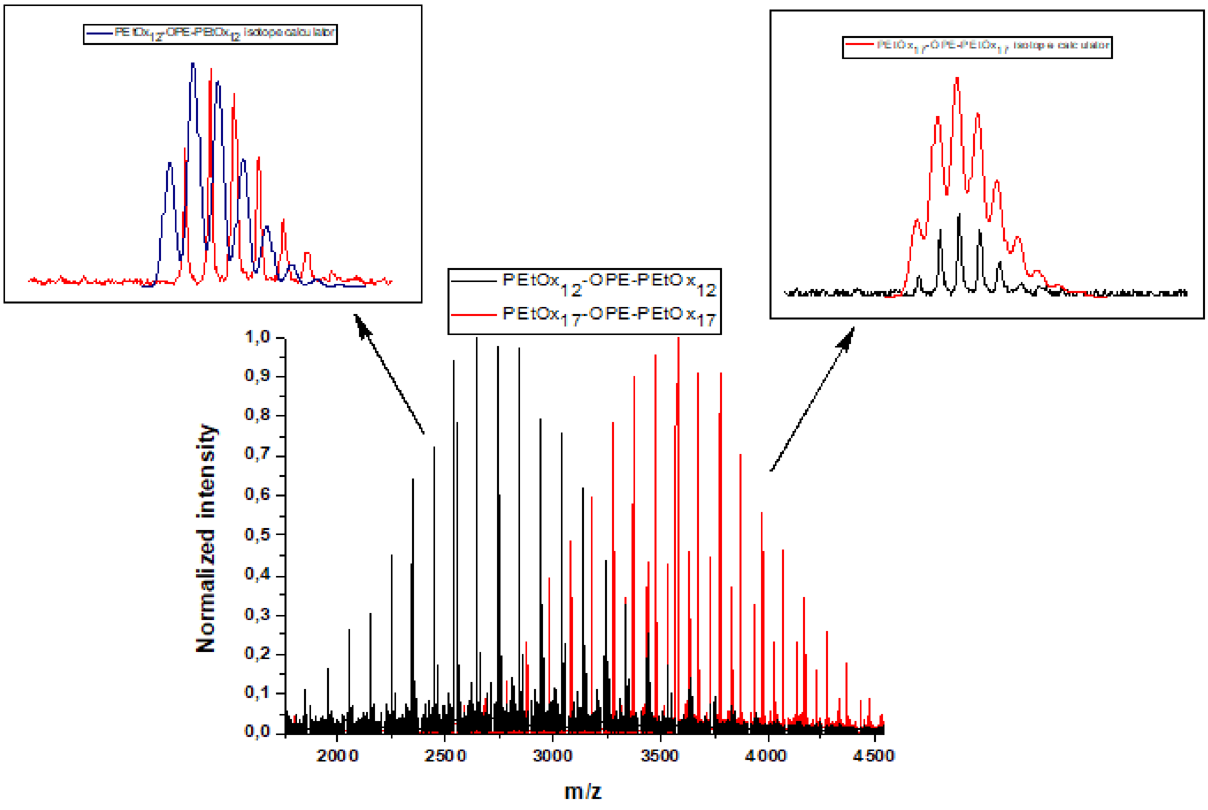

Figure S4: Characterization *via* MALDI-ToF MS (Matrix: DCTB, salt: NaCl) for $PEtOx_{12}$-OPE- $PEtOx_{12}$ (black curve) and $PEtOx_{17}$-OPE- $PEtOx_{17}$ (red curve); inset: magnification of the measured spectra (black and red curve) and calculated spectra (blue curve).

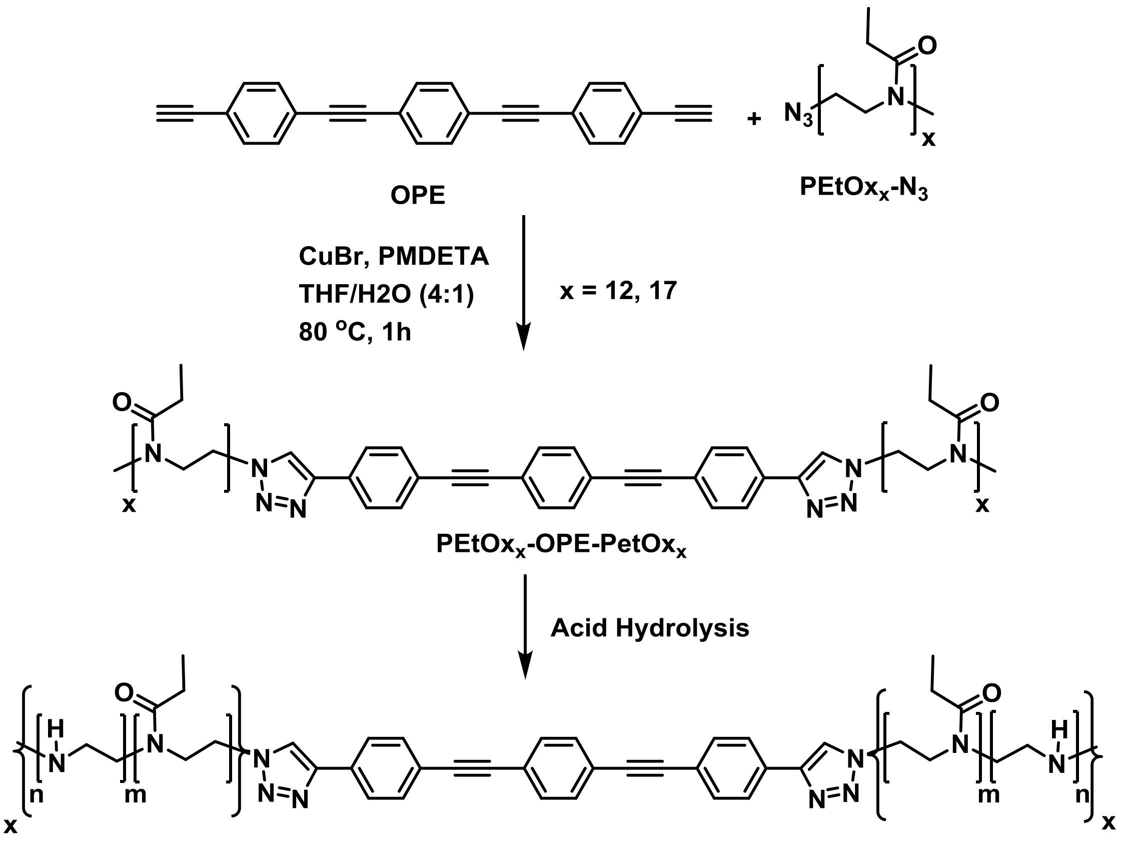

1a: DP (x) = 17, DH = 50 % (m = n = 0.5)

1b: DP (x) = 17, DH = 75 % (m = 0.25; n = 0.75)

2a: DP (x) = 12, DH = 50 % (m = n = 0.5)

2b: DP (x) = 12, DH = 75 % (m = 0.25; n = 0.75)

Scheme S1: A scheme showing synthesis of **1a-b** and **2a-b**.

Synthesis of partially cleaved (PEtOx$_m$-*co*-PEI$_n$)$_x$-OPE-(PEtOx$_m$-*co*-PEI$_n$)$_x$ (x=12, 17) (1a-b and 2a-b)

In a microwave vial, PEtOx$_x$-OPE-PEtOx$_x$ was dissolved in 6M hydrochloric acid (HCl) giving a final amide concentration of 0.48M. The resulting solutions were heated to 100 °C for 110 and 150 min, respectively. After that, the reaction solutions were directly transferred into a Float-ALyzer ® (MWCO: 100-500Da) and dialyzed against water for one day. The pure polymers were obtained after freeze-drying.

^{1}H-NMR (300 MHz, MeOD, δ): 8.0-7.5 (aromatic), 3.7–3.4 (br,–N–*CH₂*–*CH₂*–, PEtOx), 2.9-2.6 (br, –NH–*CH₂*–*CH₂*–, PEI), 2.5–2.3 (br, CO–*CH₂*–*CH₃*), 1.2–1.0 (br, CO–*CH₂*–*CH₃*) ppm.

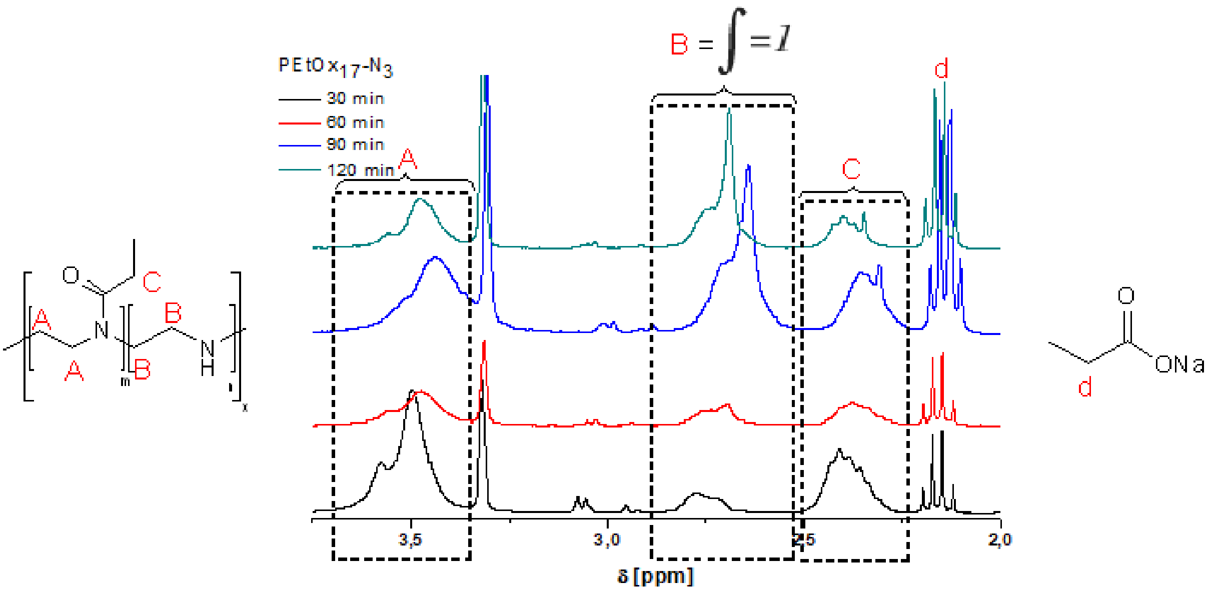

Figure S5: Comparison of NMR spectra: series of ^{1}H-NMR (300 MHz, MeOD) spectra corresponding to the hydrolysis after different reaction times of $PEtOx_{17}$-N_3 in 6M HCl at 100 °C.

Table S1: Degree of hydrolysis for PEtOx-N_3 with DP = 12, 17 after different reaction times in a microwave synthesizer at 100 °C with a constant amide concentration of 0.48M.

DP ($PEtOx_xN_3$)	t [min]	*I* ^{a} (PEtOx CH_2)	DH [%]
**12**	30	3,32	13
**12**	60	0,91	35
**12**	90	0,49	51
**12**	120	0,32	61
**17**	30	3,42	13
**17**	60	1,22	29
**17**	90	0,5	50
**17**	120	0,37	57

a) NMR (300 MHz, MeOD)

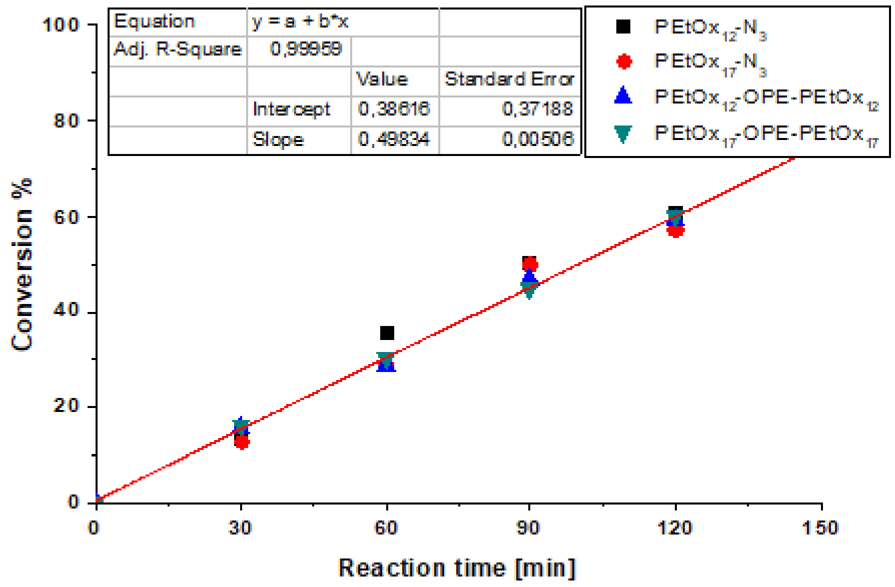

Figure S6: Conversion *vs* time plot for the acidic hydrolysis of PEtOx$_x$-N$_3$ with DP = 12, 17 and PEtOx$_x$-OPE-PEtOx$_x$ with DP = 12, 17 in a microwave synthesizer at 100 °C with a constant amide concentration of 0.48M with linear fit of PEtOx$_{17}$-OPE-PEtOx$_{17}$.

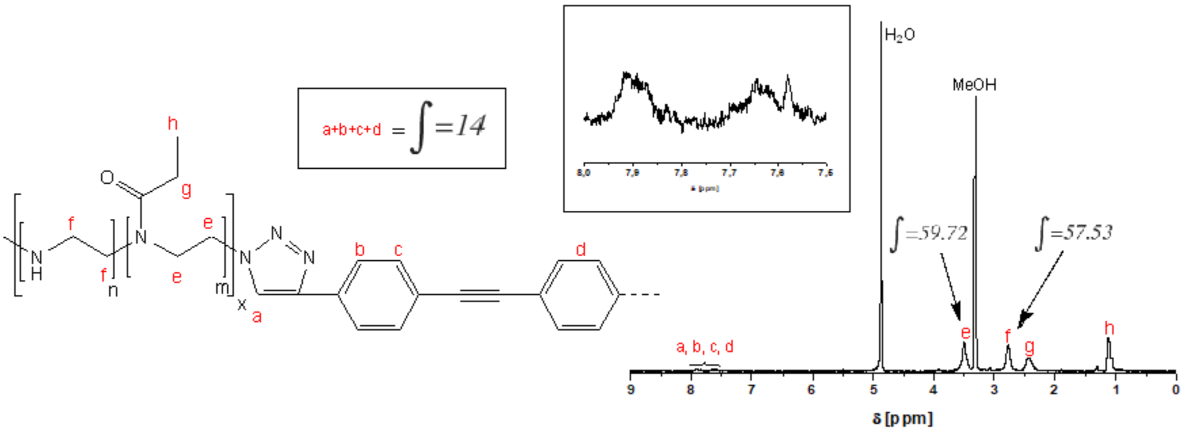

Figure S7: Characterization of P(EtOx$_{0.51}$-*co*-EI$_{0.49}$)$_{17}$-OPE-P(EtOx$_{0.51}$-*co*-EI$_{0.49}$)$_{17}$ with a DP of 17 and a degree of hydrolysis of 49 % *via* NMR (300 MHz, MeOD).

Table S2: Details for the synthesized P(EtOx$_m$-*co*-EI$_n$)$_x$-OPE-P(EtOx$_m$-*co*-EI$_n$)$_x$ with defined degrees of hydrolysis.

DP[a]	DH^{theo} [%]	DH[b] [%]	m [mg]
**12**	50	52	100
**12**	75	79	66
**17**	50	49	38
**17**	75	73	45

a) determined via NMR for the PEtOx (300 MHz, $CDCl_3$), b) determined via NMR (300 MHz, MeOD).

C. Supplementary Figures and Table

Table S3: Details for the synthesized **1a-b** and **2a-b** with defined degrees of hydrolysis.
a) NMR (300 MHz, MeOD) b) calculated from corresponding DP and DH c) calculated to 50% protonation (pka of LPEI is 7.9).[S2]

DP[a]	DH[a] [%]	$DP_{calc}^{PEtOx,b}$	$DP_{calc}^{PEI,b}$	DP[c] of protonated PEI
**1a**	49	8.7	8.3	4.2
**1b**	73	6.6	12.4	6.2
**2a**	52	5.8	6.2	3.1
**2b**	79	2.5	9.5	4.7

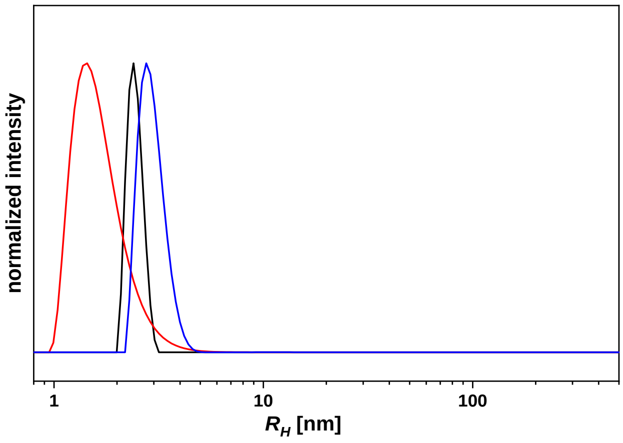

Figure S8: Number-weighted DLS CONTIN plots of **1a** at (a) pH 3 (black trace), pH 7 (red trace) and pH 11 (blue trace).

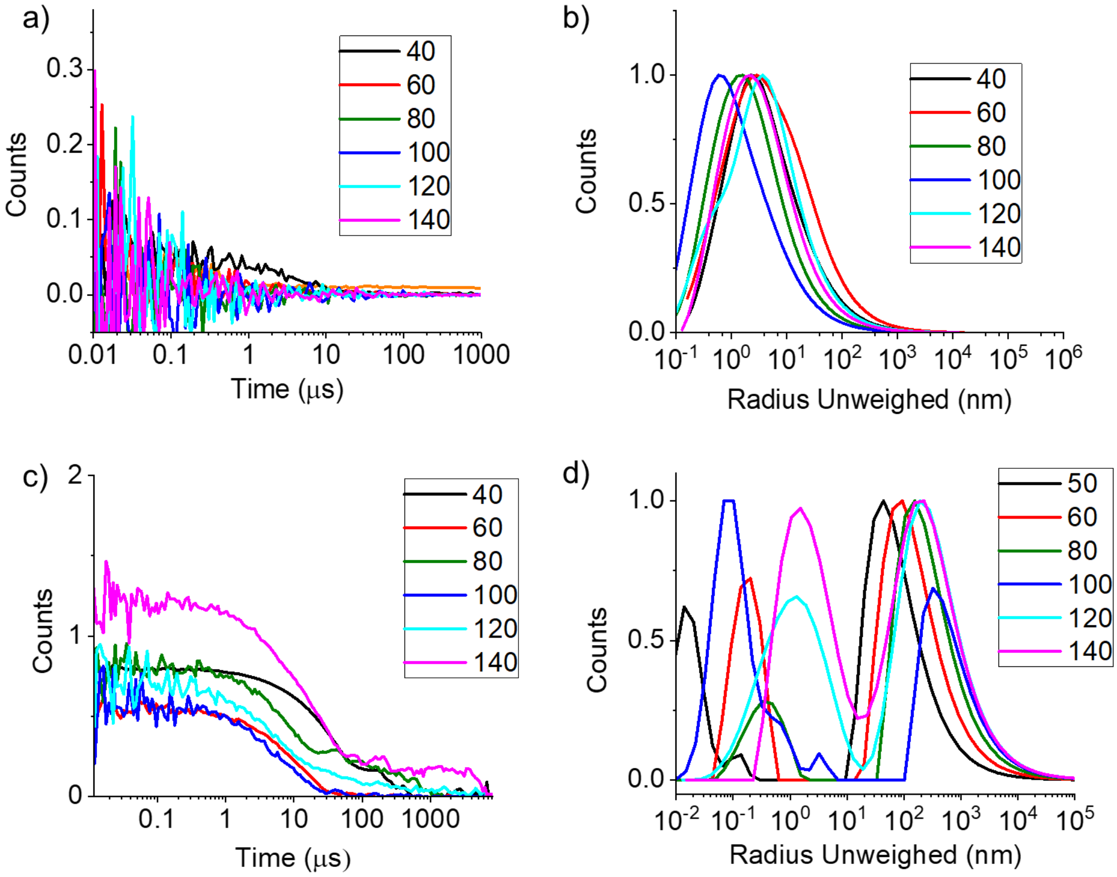

Figure S9. Angular dependent DLS studies of **1b** at pH3 (a, b) and pH 11(c, d): a,c) Correlation function and b, d) Size distribution. For **1b** at pH 3, angular-dependent dynamic light scattering (DLS) studies show one size distribution from 40-140 degrees, indicating the presence of isotropic nanostructures. On the other hand, at pH 11, two size distributions (10 nm, 100-1000 nm) were observed, which are highly dependent with the scattering angle, confirmed the anisotropic nanostructures.

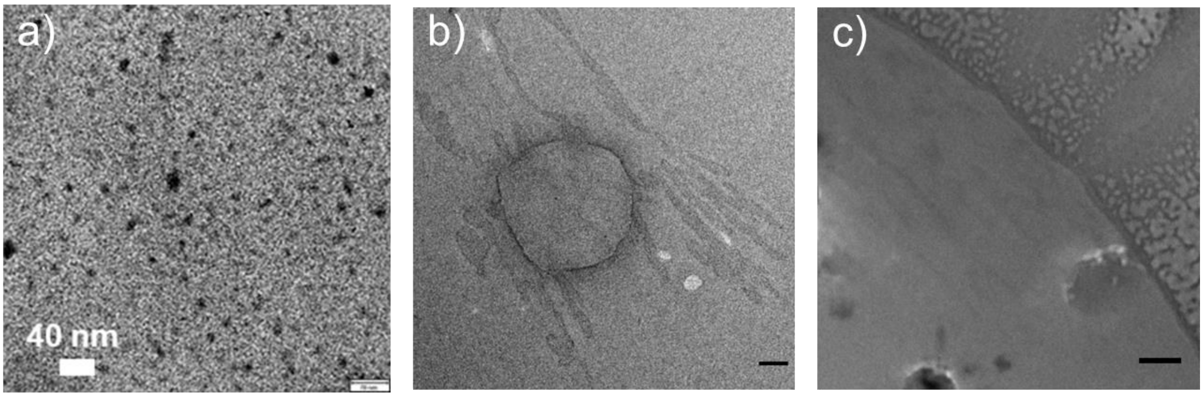

Figure S10: TEM micrograph of **1b** at pH 3 (a), pH 11 (b) and cryo-TEM micrograph of **1b** at pH 11 (c); the scale bars in b) and c) correspond to 100 nm.

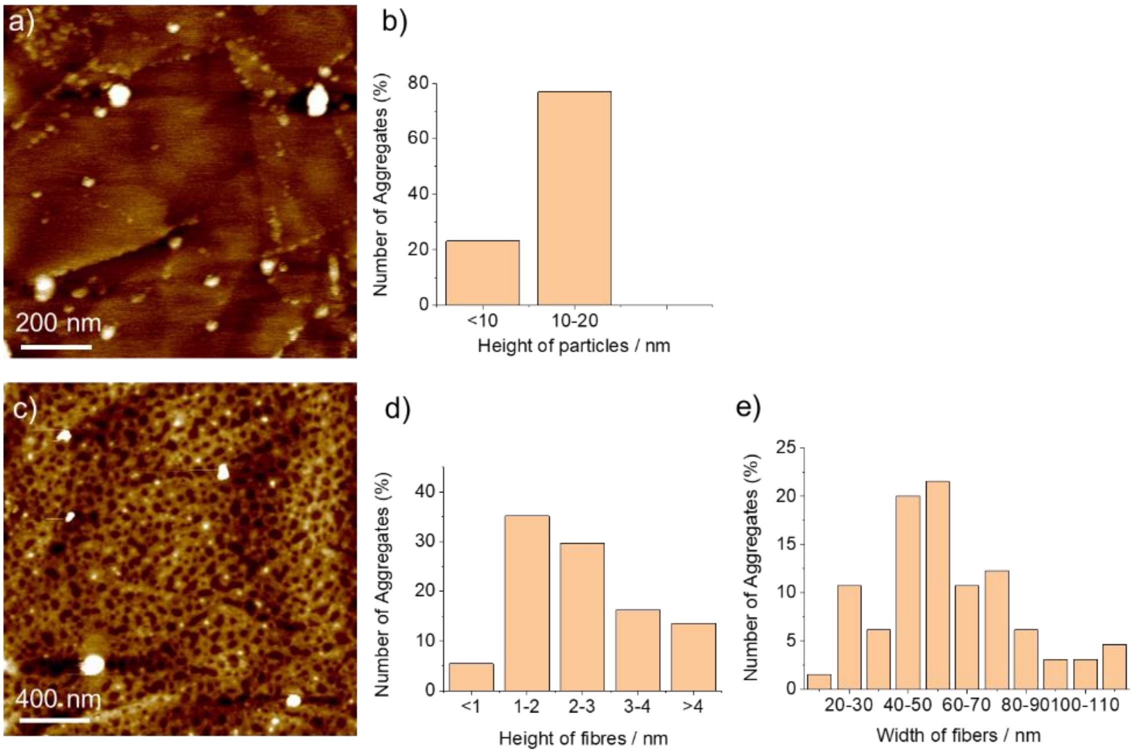

Figure S11: AFM height image of **1b** at a) pH 3 c) pH 11 on a HOPG surface ($c = 5 \times 10^{-6}$ M). Corresponding statistical analysis showing distribution of b) particles in the range of 10-20 nm, 2D nanostructures showing distribution of fibres with d) height in the range of 1-4 nm and e) width in a range of 20-120 nm.

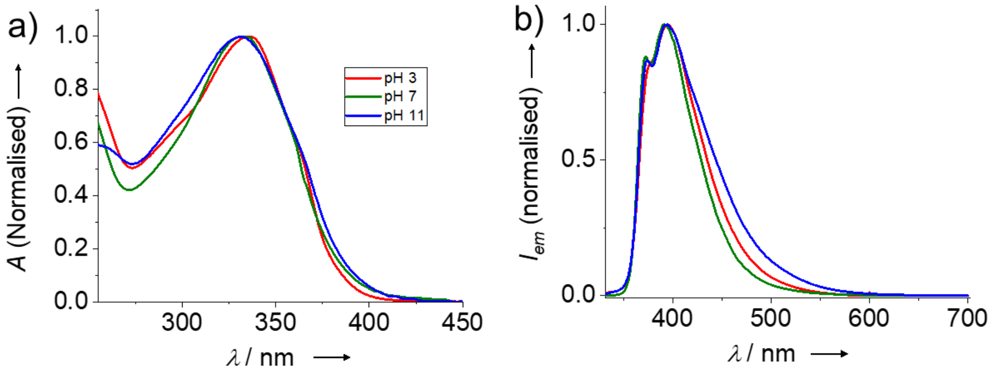

Figure S12: Normalised UV-Vis (a) and fluorescence spectra (b) of **1b** at different pH (3-red, 7-green and 11-blue) and 363 K.

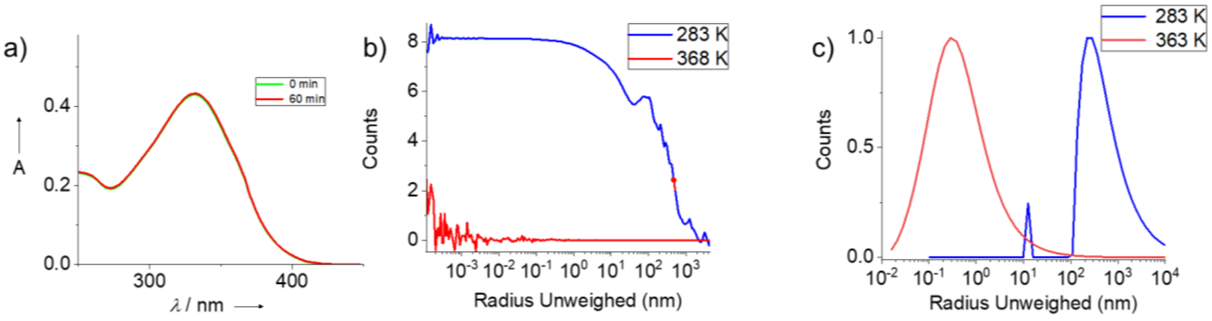

Figure S13: (a) UV-Vis spectra of **1b** at pH 11 and 368 K monitored over time (0 min- green and 60 min-red). No spectral changes occur over 60 min at 368 K, further supporting the presence of a monomeric state. Temperature-dependent DLS at 283 K (blue) and 368 K (red) of **1b** at pH 11; b) correlation function c) size distribution. The low values of hydrodynamic radii (maximum at 0.32 nm) are in accordance with the monomer species.

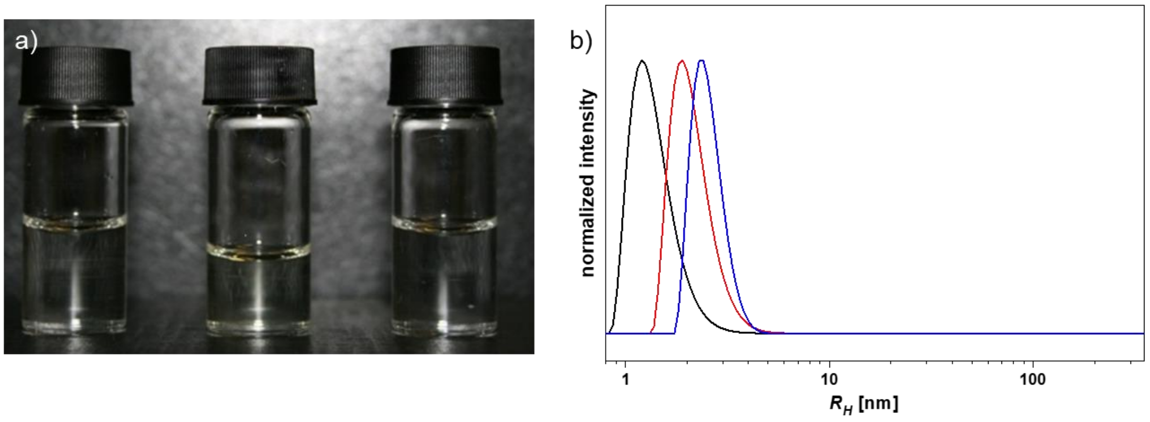

Figure S14: Photographs of the aqueous solution of **2a** at different pH (left to right - 3,7 and 11) and number-weighted DLS CONTIN plots of **2a** at pH 3 (black trace), pH 7 (red trace) and pH 11 (blue trace).

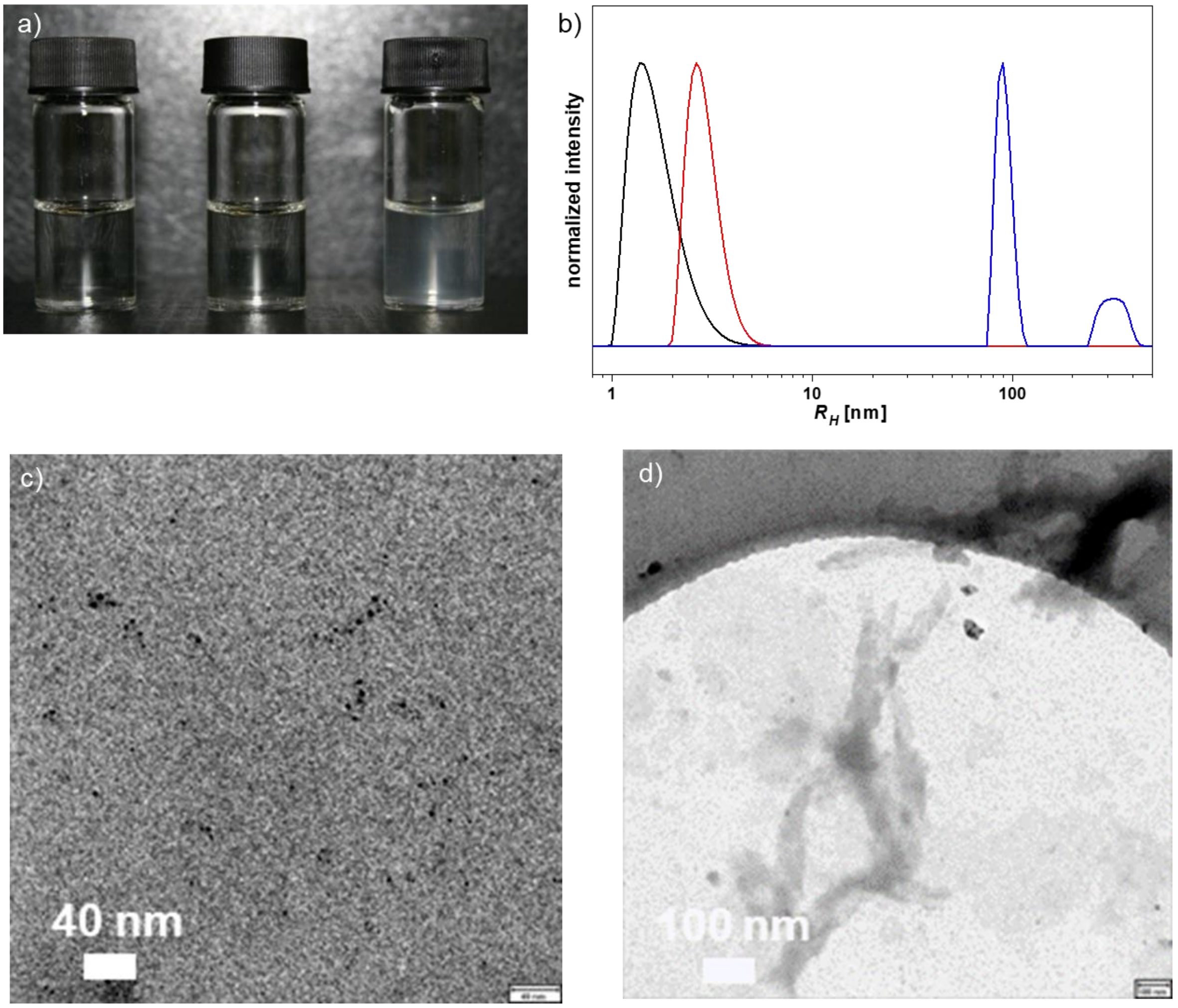

Figure S15: Photographs of the aqueous solution of **2b** at different pH (left to right - 3,7 and 11) and number-weighted DLS CONTIN plots of **2b** at pH 3 (black trace), pH 7 (red trace) and pH 11 (blue trace). TEM micrograph of **2b** at pH 3 (c) and at pH 11 (d).

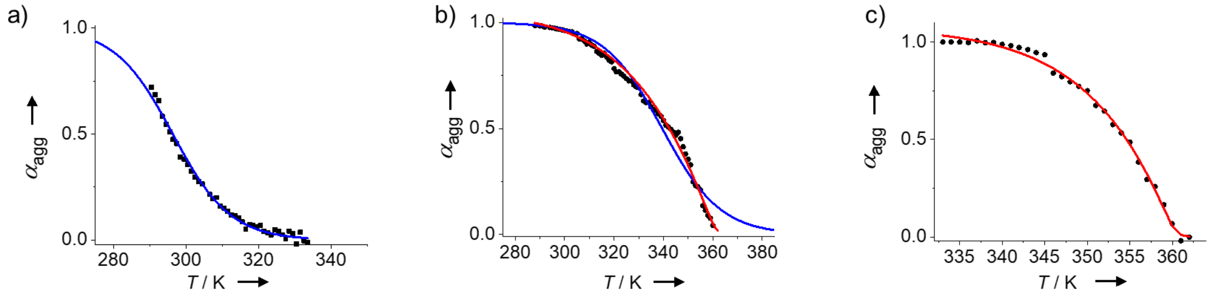

Figure S16: Cooling curves of **2b** at pH 3 (a), pH 7 (b) and pH 11 (c) obtained by monitoring UV-Vis spectral changes at 335 nm. At pH 3, a sigmoidal shape is observed, suggesting the existence of isodesmic self-assembly mechanism (blue fits in a). A cooperative mechanism is found at pH = 11 by fitting to the nucleation-elongation model (red fit in c). At pH = 7, both cooperative (red) and isodesmic (blue) fail to describe the experimental data accurately.

Table S4: Thermodynamic parameters of **1b** and **2b** at different pH values calculated using the isodesmic and nucleation-elongation model ($c = 5 \times 10^{-6}$ M). High degree of cooperativity was observed for **1b** and **2b** at pH 11, whereas at pH 3 adequate results were obtained with isodesmic fit.

Sample	K_a [M^{-1}]	T_m [K]	ΔH [kJ/mol^{-1}]	ΔS [J/mol^{-1}]	ΔG [kJ/mol^{-1}]
1b (pH 3)	3.13×10^5	310.01	-74.55	-144.82	-31.37
2b (pH 3)	1.96×10^5	296.67	-103.81	-246.87	-30.21

Sample	K_n [M^{-1}]	K_e [M^{-1}]	σ	T_e [K]	ΔH [kJ/mol^{-1}]	ΔS [J/mol^{-1}]	ΔH_{nucl} [kJ/mol^{-1}]	ΔG [kJ/mol^{-1}]
1b (pH 11)	2.1	2×10^5	1.06×10^{-5}	358.0	-64.94	-0.08	-34.09	-41.92
2b (pH 11)	70.9	2×10^5	3.5×10^{-4}	360.0	-123.58	-0.24	-23.78	-51.49

References:

S1. T. Rudolph, N. Kumar Allampally, G. Fernandez, F. H. Schacher, *Chem. Eur. J.* **2014**, *20*, 13871.

S2. B. Brissault, A. Kichler, C. Guis, C. Leborgne, O. Danos, H. Cheradame, *Bioconjugate Chem.* **2003**, *14*, 581.

S3. D. V. Pergushov, A. H. E. Müller, F. H. Schacher, *Chem. Soc. Rev.* **2012**, *41*, 6888-6901.
